# Enhanced Production of Bovine Chymosin by Autophagy Deficiency in the Filamentous Fungus *Aspergillus oryzae*

**DOI:** 10.1371/journal.pone.0062512

**Published:** 2013-04-29

**Authors:** Jaewoo Yoon, Takashi Kikuma, Jun-ichi Maruyama, Katsuhiko Kitamoto

**Affiliations:** Department of Biotechnology, The University of Tokyo, Tokyo, Japan; University of Missouri, United States of America

## Abstract

*Aspergillus oryzae* has been utilized as a host for heterologous protein production because of its high protein secretory capacity and food-safety properties. However, *A. oryzae* often produces lower-than-expected yields of target heterologous proteins due to various underlying mechanisms, including degradation processes such as autophagy, which may be a significant bottleneck for protein production. In the present study, we examined the production of heterologous protein in several autophagy (*Aoatg*) gene disruptants of *A. oryzae*. We transformed *A. oryzae* gene disruptants of *Aoatg1*, *Aoatg13*, *Aoatg4*, *Aoatg8*, or *Aoatg15*, with a bovine chymosin (CHY) expression construct and found that the production levels of CHY increased up to three fold compared to the control strain. Notably, however, conidia formation by the *Aoatg* gene disruptants was significantly reduced. As large amounts of conidia are necessary for inoculating large-scale cultures, we also constructed *Aoatg* gene-conditional expression strains in which the promoter region of the *Aoatg* gene was replaced with the thiamine-controllable *thiA* promoter. Conidiation by the resultant transformants was clearly enhanced in the absence of thiamine, while autophagy remained repressed in the presence of thiamine. Moreover, these transformants displayed increased CHY productivity, which was comparable to that of the *Aoatg* gene disruptants. Consequently, we succeeded in the construction of *A. oryzae* strains capable of producing high levels of CHY due to defects in autophagy. Our finding suggests that the conditional regulation of autophagy is an effective method for increasing heterologous protein production in *A. oryzae*.

## Introduction

The filamentous fungus *Aspergillus oryzae* (Ahlburg) Cohn has the ability to secrete large amounts of proteins and it has been safely used in the preparation of fermented foods for over a thousand year in Japan [1]. Due to these merits, *A. oryzae* is generally considered to be an outstanding host for the production of heterologous proteins [2], [3]. However, when heterologous proteins derived from higher organisms are expressed in *A. oryzae*, lower-than-desired yields of the target protein are often obtained [4]–[7]. These reduced yields are thought to result from bottlenecks in the transcription, translation, and secretory pathways of *A. oryzae*. In our previous research, we observed that the expression of a mutant α-amylase protein with defective disulfide bonds in *A. oryzae* resulted in accumulation of the protein in the endoplasmic reticulum (ER) [8]. Furthermore, we confirmed that autophagy delivers the misfolded secretory proteins from the ER to vacuoles [9]. Based on these observations, autophagy appears to be a significant process adversely affecting the production of heterologous proteins in *A. oryzae*.

Autophagy, which is a highly conserved intracellular degradation pathway in eukaryotes, functions as a survival mechanism under nutrient starvation conditions by recycling intracellular components [10]. The autophagic process consists of several sequential steps: 1) induction, 2) autophagosome formation, 3) fusion of autophagosomes to lysosomes/vacuoles, and 4) degradation of autophagic bodies [11], [12]. In addition to nutrient recycling, autophagy plays important roles in cell development and differentiation, immune responses, and cell death, and has also been shown to participate in various diseases including cancer [13]. Thus, autophagy is an extremely important system in eukaryotes; however, no studies have examined the mechanisms by which autophagy affects the production of recombinant proteins.

Our group previously demonstrated that decreasing protease activity in *A. oryzae* through the disruption of ten protease genes led to increased productivity of heterologous proteins [14]. Moreover, disruption of the gene coding for the vacuolar protein sorting (Vps) receptor AoVps10 led to decreased transportation of recombinant proteins to vacuoles via the Vps pathway and increased secretion of recombinant proteins in the culture medium [15]. This finding suggests that repression of vacuolar degradation may be effective in enhancing the productivity of heterologous proteins.

In the present study, the effect of disruption of genes involved in autophagy on heterologous protein production in *A. oryzae* was examined. *A. oryzae* disruptants were constructed for five genes related to autophagy: *Aoatg1*, encoding a kinase involved autophagy induction [16]; *Aoatg13*, encoding a component of the AoAtg1 complex [17], [18]; *Aoatg4* and *Aoatg8*, which are essential genes for the formation of autophagosomes and membrane fusion [19]; and *Aoatg15*, encoding a lipase required for the breakdown of autophagic bodies [20]–[22]. Using the *A. oryzae* gene disruptants, the production of bovine chymosin (CHY) was measured as a reporter heterologous protein. This is the first report demonstrating that the disruption of autophagy-related genes in a filamentous fungus enhances the production levels of heterologous proteins.

## Materials and Methods

### Strains, Media, and Transformation

*A. oryzae* wild-type strain RIB40 and strain NSPlD1, which has a highly efficient gene-targeting background (*niaD* ^−^ *sC* ^−^ Δ*pyrG* Δ*ligD*) [23], were used as a DNA donor and transformation host, respectively. The strains used and generated in this study are listed in Table 1. Potato Dextrose (PD) agar (Nissui Phamaceutical) medium was used for the growth and maintenance of all strains. PD agar containing 10 µM thiamine was used for harvesting conidia from *Aoatg* gene-conditional expression strains. M medium [0.2% NH_4_Cl, 0.1% (NH_4_)_2_SO_4_, 0.05% KCl, 0.05% NaCl, 0.1% KH_2_PO_4_, 0.05% MgSO_4_·7H_2_O, 0.002% FeSO_4_·7H_2_O, and 2% glucose, pH 5.5] supplemented with 0.15% methionine (M+Met) was used as a selective medium for replacing the *Aoatg* promoter. 5×DPY medium [10% dextrin, 5% polypeptone, 2.5% yeast extract, 0.5% KH_2_PO_4_, and 0.05% MgSO_4_·7H_2_O, pH 5.5] was used for CHY production. Czapek-Dox (CD) medium (0.3% NaNO_3_, 0.2% KCl, 0.1% KH_2_PO_4_, 0.05% MgSO_4_·7H_2_O, 0.002% FeSO_4_·7H_2_O, and 2% glucose, pH 5.5) supplemented with 0.0015% methionine was used for *niaD*-based plasmid integration. *Escherichia coli* DH5α [*supE44* Δ*lacU169* (*Φ80 lacZ* Δ*M15*) *hsdR17 recA1 endA1 gyrA96 thi-1 relA1*] was used for DNA manipulation. *A. oryzae* was transformed according to a previously reported method [1].

**Table 1:** List of strains used in the study.

Strain name	Parental strain	Genotype	Reference
RIB40	Wild-type		
NSRKu70-1-1	NSAR1	*niaD^−^ sC^−^ adeA^-^* Δ*argB*::*adeA^−^* Δ*ku70*::*argB*	[36]
NSKu70-AA	NSRKu70-1-1	*niaD^−^ sC^−^ adeA^−^* Δ*argB*::*adeA^−^* Δ*ku70*::*argB adeA*	[37]
NSKu70-Aoatg1	NSRKu70-1-1	*niaD^−^ sC^−^ adeA^−^* Δ*argB*::*adeA^−^* Δ*ku70*::*argB* Δ*Aoatg1*::*adeA*	[30]
NSKu70-ΔAoatg4-2	NSRKu70-1-1	*niaD^−^ sC^−^ adeA^−^* Δ*argB*::*adeA^−^* Δ*ku70*::*argB* Δ*Aoatg4*::*adeA*	[29]
ΔAoatg8	NSR13	*niaD^−^ sC^−^ adeA^−^* Δ*Aoatg8*::*adeA*	[28]
NSKu70-ΔAoatg13	NSRKu70-1-1	*niaD^−^ sC^−^ adeA^−^* Δ*argB*::*adeA^-^* Δ*ku70*::*argB* Δ*Aoatg13*::*adeA*	[29]
NSKu70-ΔAoatg15-10-1	NSRKu70-1-1	*niaD^−^ sC^−^ adeA^−^* Δ*argB*::*adeA^-^* Δ*ku70*::*argB* Δ*Aoatg15*::*adeA*	[29]
SKu70-AA-AKC1	NSKu70-AA	*niaD^−^*::pgAKCN[P*amyB*::*amyB*-*kex2*-*CHY*::T*amyB*::*niaD*]*sC^−^ adeA^−^* Δ*argB*::*adeA^−^* Δ*ku70*::*argB* Δ*Aoatg1*::*adeA*	[27]
SKu70-ΔAoatg1-AKC2	NSKu70-ΔAoatg1	*niaD^−^*::pgAKCN[P*amyB*::*amyB*-*kex2*-*CHY*::T*amyB*::*niaD*] *sC^−^ adeA^−^* Δ*argB*::*adeA^-^* Δ*ku70*::*argB* Δ*Aoatg1*::*adeA*	This study
SKu70-ΔAoatg4-AKC1	NSKu70-ΔAoatg4-2	*niaD^−^*::pgAKCN[P*amyB*::*amyB*-*kex2*-*CHY*::T*amyB*::*niaD*] *sC* ***^−^*** * adeA^−^* Δ*argB*::*adeA^−^* Δ*ku70*::*argB* Δ*Aoatg4*::*adeA*	This study
ΔAoatg8-AKC1	ΔAoatg8	*niaD^−^*::pgAKCN[P*amyB*::*amyB*-*kex2*-*CHY*::T*amyB*::*niaD*] *sC^−^ adeA^−^* Δ*Aoatg8*::*adeA*	This study
SKu70-ΔAoatg13-AKC1	NSKu70-ΔAoatg13	*niaD^−^*::pgAKCN[P*amyB*::*amyB*-*kex2*-*CHY*::T*amyB*::*niaD*] *sC^−^ adeA^−^* Δ*argB*::*adeA^−^* Δ*ku70*::*argB* Δ*Aoatg13*::*adeA*	This study
SKu70-ΔAoatg15-AKC2	NSKu70-ΔAoatg15-10-1	*niaD^−^*::pgAKCN[P*amyB*::*amyB*-*kex2*-*CHY*::T*amyB*::*niaD*] *sC^−^ adeA^−^* Δ*argB*::*adeA^−^* Δ*ku70*::*argB* Δ*Aoatg15*::*adeA*	This study
NSPlD1	NSR-ΔlD2	*niaD^−^ sC^−^ adeA^−^* Δ*argB*::*adeA^−^* Δ*ligD*::*argB pyrG^−^*	[23]
NSlD1	NSPlD1	*niaD^−^ sC^−^ adeA^−^* Δ*argB*::*adeA^−^* Δ*ligD*::*argB*	[14]
SlD-AKC1	NSlD1	*niaD^−^*::pgAKCN[P*amyB*::*amyB-kex2-CHY*::T*amyB*::*niaD] sC^−^ adeA^−^* Δ*argB*::*adeA^-^* Δ*ligD*::*argB pyrG^-^*	[14]
NSlD-PtA1	NSPlD1	*niaD^−^ sC^−^ adeA^−^* Δ*argB*::*adeA^−^* Δ*ligD*::*argB pyrG^−^*::[P*Aoatg1*::*pyrG-*P*thiA*::*Aoatg1*]	This study
NSlD-PtA4	NSPlD1	*niaD^−^ sC^−^ adeA^−^* Δ*argB*::*adeA^−^* Δ*ligD*::*argB pyrG^−^*::[P*Aoatg4*::*pyrG*-P*thiA*::*Aoatg4*]	This study
NSlD-PtA8	NSPlD1	*niaD^−^ sC^−^ adeA^−^* Δ*argB*::*adeA^−^* Δ*ligD*::*argB pyrG^−^*::[P*Aoatg8*::*pyrG-*P*thiA*::*Aoatg8*]	This study
NSlD-PtA15	NSPlD1	*niaD^−^ sC^−^ adeA^-^* Δ*argB*::*adeA^-^* Δ*ligD*::*argB pyrG^-^*::[P*Aoatg15*::*pyrG-*P*thiA*::*Aoatg15*]	This study
SlD-PtA1-AKC	NSlD-PtA1	*niaD^−^*::pgAKCN[P*amyB*::*amyB-kex2-CHY*::T*amyB*::*niaD*] *sC^−^ adeA^−^* Δ*argB*::*adeA^-^* Δ*ligD*::*argB pyrG^−^*::[*Aoatg1*::*pyrG-*P*thiA*::*Aoatg1*]	This study
SlD-PtA4-AKC	NSlD-PtA4	*niaD^−^*::pgAKCN[P*amyB*::*amyB-kex2-CHY*::T*amyB*::*niaD*] *sC^-^ adeA^-^* Δ*argB*::*adeA^-^* Δ*ligD*::*argB pyrG^−^*::[P*Aoatg4*::*pyrG*-P*thiA*::*Aoatg4*]	This study
SlD-PtA8-AKC	NSlD-PtA8	*niaD^−^*::pgAKCN[P*amyB*::*amyB-kex2-CHY*::T*amyB*::*niaD*] *sC^−^ adeA^−^* Δ*argB*::*adeA^−^* Δ*ligD*::*argB pyrG^-^*::[P*Aoatg8*::*pyrG-*P*thiA*::*Aoatg8*]	This study
SlD-PtA15-AKC	NSlD-PtA15	*niaD^−^*::pgAKCN[P*amyB*::*amyB-kex2-CHY*::T*amyB*::*niaD*] *sC^−^ adeA^−^* Δ*argB*::*adeA^−^* Δ*ligD*::*argB pyrG^−^*::[P*Aoatg15*::*pyrG-*P*thiA*::*Aoatg15*]	This study

### Construction of Conditional Expression Strains

Plasmid pgPtA1 was constructed to replace the *Aoatg1* promoter with the *thiA* promoter using the MultiSite Gateway cloning system (Invitrogen). The upstream and N-terminal 1.5-kb regions of the *Aoatg1* gene were amplified by PCR using the primer pairs *att*B4-PAoatg1-F and *att*B1-PAoatg1-R, and *att*B2-Aoatg1-F and *att*B3-Aoatg1-R, respectively (Table 2). The amplified *att*B-flanked upstream and *Aoatg1* fragments were introduced into pDNOR^TM^P4-P1R and pDNOR^TM^P2R-P3, respectively, using the Gateway BP Clonase Reaction Mix (Invitrogen), generating the Entry Clone plasmids pg5′upAoatg1 and pg3′Aoatg1, respectively. The plasmids pg5′upAoatg1 and pg3′Aoatg1, an Entry Clone plasmid containing the *A. oryzae pyrG* gene as a selective marker and the *thiA* promoter, and the Destination vector pDEST^TM^R4-R3 (Invitrogen) were mixed and then subjected to the Gateway LR reaction using the Gateway LR Clonase reaction mix (Invitrogen) to generate pgPtA1. Using plasmid pgPtA1 as a template, the sequence containing the replacement cassette, which consisted of the upstream region of *Aoatg1* (1.5 kb), *pyrG* gene (2.0 kb), *thiA* promoter (1.3 kb), and N-terminal region of the *Aoatg1* gene (1.5 kb), was amplified by PCR with the primers *att*B4-upAoatg1-F and *att*B3-Aoatg1-R, and then transformed into *A. oryzae* NSRKu70-1-1. Recombination was confirmed by Southern blotting using a 1.5-kb fragment of the *Aoatg1* gene upstream region as a probe, which was generated by PCR with the primers *att*B4-upAoatg1-F and *att*B1-upAoatg1-R (Figure S1).

**Table 2:** List of primers used in this study.

Primer name	Sequence (5'-3')
*att*B4-PAoatg1-F	GGGGACAACTTTGTATAGAAAAGTTGCCTTTCTCCTCTCTACCTTG
*att*B1-PAoatg1-R	GGGGACTGCTTTTTTGTACAAACTTGCCTAAGAGGAGCGTCTACAA
*att*B2-Aoatg1-F	GGGGACAGCTTTCTTGTACAAAGTGGATGTCGTCTTCACACCACAG
*att*B3-Aoatg1-R	GGGGACAACTTTGTATAATAAAGTTGCCGTAGCGTTTCGATCTACA
*att*B4-PAoatg4-F	GGGGACAACTTTGTATAGAAAAGTTGTCATCTTCACTAGAGCTGTCACG
*att*B1-PAoatg4-R	GGGGACTGCTTTTTTGTACAAACTTGTATGGTTGGCTGGGTAGTGAA
*att*B2-Aoatg4-F	GGGGACAGCTTTCTTGTACAAAGTGGATGAACAGTGTAGACATAGGGCG
*att*B3-Aoatg4-R	GGGGACAACTTTGTATAATAAAGTTGCCCTTCTGCCTTGGTTGTAAGTA
*att*B4-PAoatg8-F	GGGGACAACTTTGTATAGAAAAGTTGTCTGAAAGTTGCAAGGTGCG
*att*B1-PAoatg8-R	GGGGACTGCTTTTTTGTACAAACTTGATTGATGGATCGAATCAGTTAATGG
*att*B2-Aoatg8-F	GGGGACAGCTTTCTTGTACAAAGTGGATGCGCTCCAAGTTCAAG
*att*B3-Aoatg8-R	GGGGACAACTTTGTATAATAAAGTTGTTTTCACTACTTATTTTCAATTACC
*att*B4-PAoatg15-F	GGGGACAACTTTGTATAGAAAAGTTGCCGACTAGAAGTAATGTGGC
*att*B1-PAoatg15-R	GGGGACTGCTTTTTTGTACAAACTTGATTTGTTGAGAGGTACCTTATACTTC
*att*B2-Aoatg15-F	GGGGACAGCTTTCTTGTACAAAGTGGATGATTATTTCAAATGCTCTTCTGGG
*att*B3-Aoatg15-R	GGGGACAACTTTGTATAATAAAGTTGTCAGGGTGGCGTGGTGAT

The underlined sequences indicate the MultiSite Gateway *att*B recombination sites.

The plasmids pgPtA4, pgPtA8, and pgPtA15 for replacement of the *Aoatg4*, *Aoatg8*, and *Aoatg15* promoters, respectively, were constructed by the identical method used for replacement of the *Aoatg1* promoter. The 1.0-kb upstream region of the *Aoatg4* gene and a 1.0-kb fragment containing the *Aoatg4* gene were amplified by PCR using the primer pairs *att*B4-PAoatg4-F and *att*B1-PAoatg4-R, and *att*B2-Aoatg4-F and *att*B3-Aoatg4-R, respectively (Table 2). The 1.0-kb upstream region of the *Aoatg8* gene and a 0.9-kb region containing the *Aoatg8* gene were amplified by PCR using the primer pairs *att*B4-PAoatg8-F and *att*B1-PAoatg8-R, and *att*B2-Aoatg8-F and *att*B3-Aoatg8-R, respectively (Table 2). The 1.5-kb upstream region of the *Aoatg15* gene and a 1.8-kb region containing the *Aoatg15* gene were amplified by PCR using the primer pairs *att*B4-PAoatg15-F and *att*B1-PAoatg15-R, and *att*B2-Aoatg15-F and *att*B3-Aoatg15-R, respectively (Table 2). All primers were based on the sequence in the *A. oryzae* genome database (http://www.bio.nite.go.jp/dogan/project/view/AO). The PCR reactions were performed using the genomic DNA of *A. oryzae* RIB40 [24] as a template.

### Southern Blot Analysis

The *A. oryzae* autophagy gene-conditional expression strains and strains expressing CHY were analyzed by Southern blot analysis. Briefly, after electrophoresis, the digested genomic DNAs of each strain were transferred onto a Hybond N+ membrane (GE Healthcare). The Enhanced Chemiluminescence (ECL) Direct Nucleic Acid Labeling and Detection System (GE Healthcare) and a LAS-4000miniEPUV luminescent image analyzer (Fuji Photo Film) were used for detection.

### Western Blot Analysis

CHY-expressing transformants were cultured in 20 ml 5×DPY (pH 5.5) medium at 30°C for 4 days. Four microliters of the culture supernatant was subjected to sodium dodecyl sulfate-polyacrylamide gel electrophoresis and the separated proteins were then transferred onto a cellulose nitrate membrane (Immobilon-NC; Millipore) using a semi-dry blotting system (Nihon Eido). The membrane was immunostained using a polyclonal rabbit serum against CHY (kindly gifted by Dr. Tsuchiya [25]), followed by treatment with antirabbit immunoglobulin G labeled with horseradish peroxidase (Cotalog No. PI-1000; Vector Laboratories), and bands were visualized using the ECL Advance™ Western Blotting Detection kit (GE Healthcare).

### Measurement of CHY Production Yield

The number of conidia harvested from PD agar plates counted using a hemocytometer under the microscope. Approximately 2×10^5^ conidia of the CHY-expressing transformant were inoculated into 20 ml 5×DPY medium (pH 5.5) and thiamine-supplemented 5×DPY medium (pH 5.5), and were then cultured at 30°C for 3–6 days. CHY activity in the culture supernatant was measured by a modification of a method of Foltmann [26] as described previously by Yoon et al. [27]. Briefly, culture supernatant (100 µl) was collected every 24 h and then mixed with 1 ml of a 12% skim milk solution containing 10 mM CaCl_2_. During the milk-clotting reaction, the mixture was shaken (60 strokes per minute) at room temperature. The time point at which the thin film of milk began to form visible particles was designated as the clotting time. The CHY production level (mg/L) in each sample was estimated from a standard curve of clotting time versus CHY protein concentration that was generated using authentic CHY (Sigma).

### Statistics

Microsoft Excel was used as a tool for statistics including means comparisons, standard deviation analyses and t-tests for significance.

## Results

### Effects of *A. oryzae* Autophagy (*Aoatg*) Gene Disruption on CHY Production

**Figure 1:**
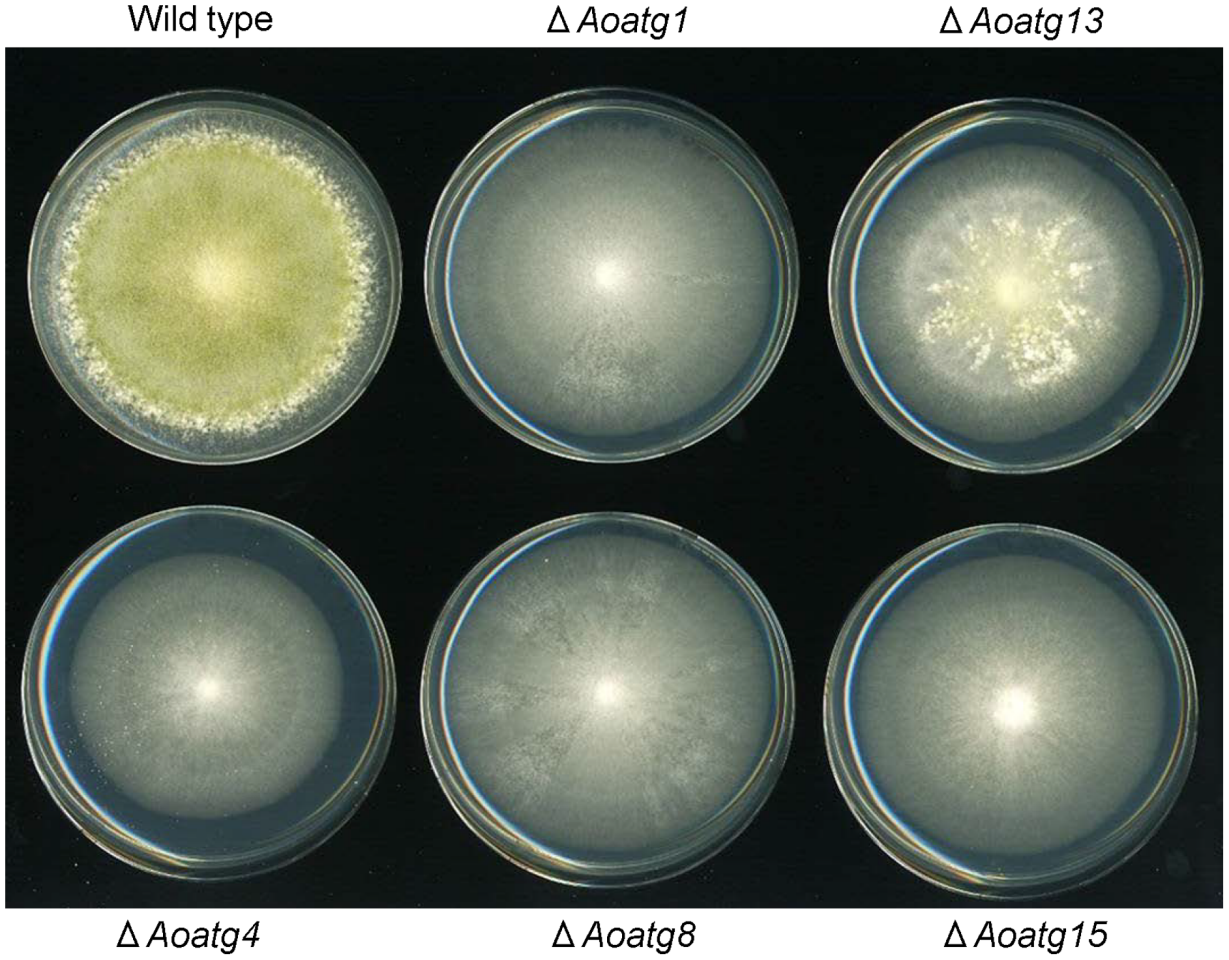
Phenotype of the *A.oryzae* autophagy gene disruptants. Images of the *Aoatg* disruptants expressing CHY after growth on PD agar plates for 4 days at 30°C.

To examine the effect of autophagy impairment on heterologous protein production in *A. oryzae*, gene disruptants of *Aoatg1*, *Aoatg13*, *Aoatg4*, *Aoatg8*, and *Aoatg15*, which were previously constructed in our laboratory [28]–[30], were modified to produce CHY by transformation of a CHY expression plasmid fused with AmyB [27]. The plasmid for CHY expression was introduced into the control strain and the autophagy gene disruptants using the *niaD* gene as the selectable marker. The transformants possessing a single copy of the plasmid, which was integrated homologously at the *niaD* locus were identified by Southern blot analysis (data not shown). When the *Aoatg1*, *Aoatg4*, *Aoatg8*, and *Aoatg15* disruptants expressing CHY were cultured for 5 days at 30°C on PD agar medium, conidia formation was clearly impaired (Figure 1). Under the same conditions, the *Aoatg13* disruptant formed a modest conidia (Figure 1).

**Figure 2:**
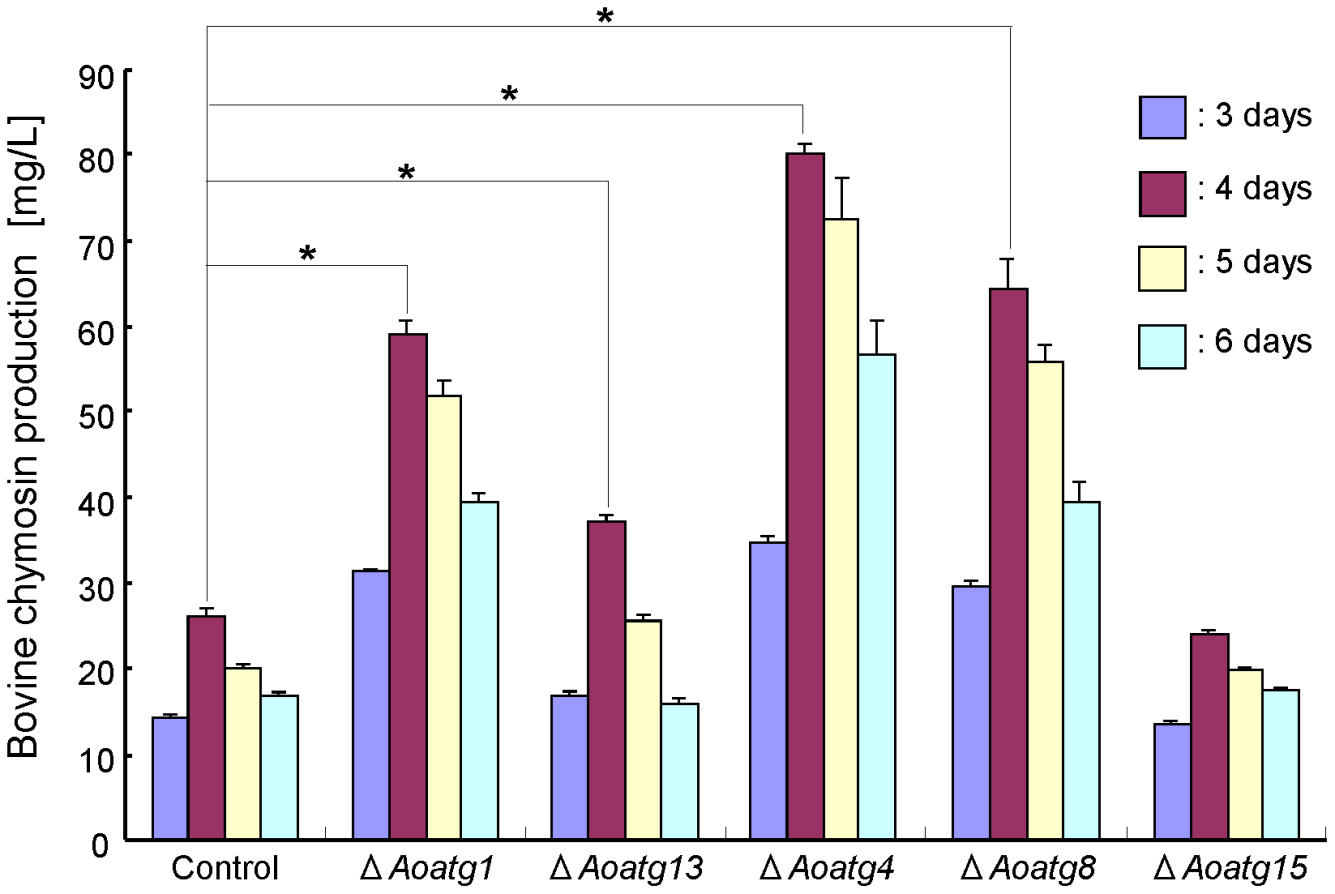
Extracellular bovine chymosin (CHY) production by *A. oryzae* autophagy gene disruptants. Approximately 2×10^5^ conidia of each strain were inoculated into 20 ml 5×DPY medium (pH 5.5) and the CHY activity in the culture supernatant was measured daily after 3–6 days of growth at 30°C. Three experiments were performed, and the values of the average and standard deviations are represented (*p<0.01, Student’s *t* test).

The production levels of CHY by the autophagy gene disruptants were estimated by measuring the milk-clotting activity of the culture supernatant (Figure 2). Each disruptant produced the highest level of CHY after 4 days of cultivation in 5×DPY medium (Figure 2). Compared to the control strain (SlD-AKC1), the production of CHY by the *Aoatg1*, *Aoatg4*, and *Aoatg8* disruptants was increased by 2.3, 3.1, and 2.5 fold, respectively. In contrast, the *Aoatg13* disruptant showed only a slight increase (1.4 fold) in CHY production. Additionally, no significant increase in CHY production by the *Aoatg15* disruptant was observed.

### Construction of *Aoatg* Gene-conditional Expression Strains

Disruption of autophagy in *A. oryzae* has almost no influence on growth on a nutrient-rich medium, but markedly reduces conidia formation. Therefore, we constructed four *Aoatg* gene-conditional expression strains by replacing the *Aoatg1*, *Aoatg4*, *Aoatg8*, and *Aoatg15* promoters with the *thiA* promoter, whose activity can be controlled by the external concentration of thiamine. The integration of the *thiA* promoter was confirmed by Southern blotting (Figure S1). Genes under control of the *thiA* promoter are induced in the absence of thiamine, and have reduced expression levels in the presence of thiamine [31].

**Figure 3:**
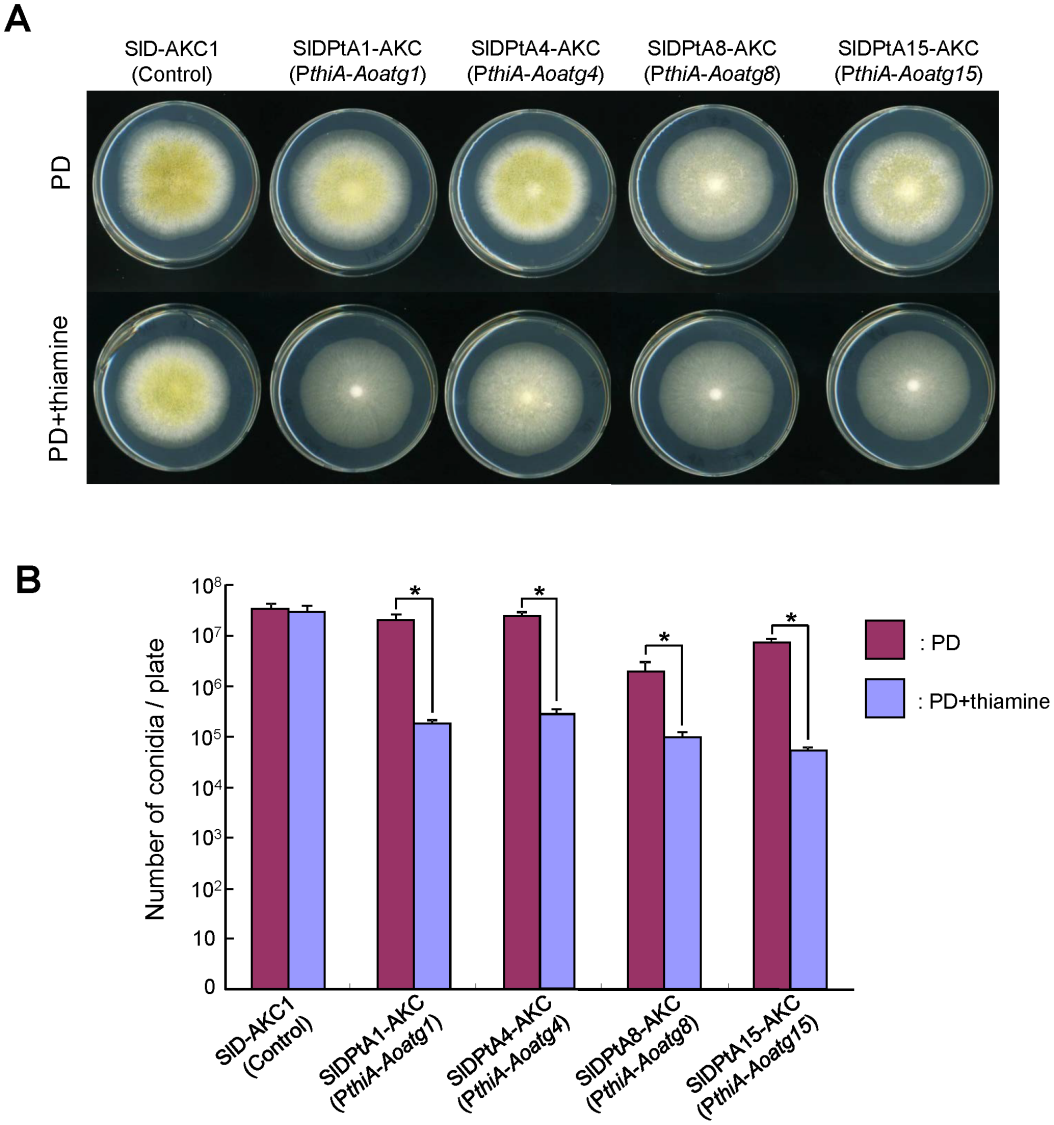
Conidiation in autophagy gene-conditional expression strains. (A) Images of the *Aoatg* conditional expression strains after growth on PD agar plates supplemented with or without thiamine for 4 days at 30°C. (B) The number of conidia formed per plate are shown for each strain under the indicated conditions. Three experiments were performed, and the values of the average and standard deviations are represented (*p<0.01, Student’s *t* test).

To examine conidia formation by the four *Aoatg* gene-conditional expression strains, each strain was cultured for 4 days on PD agar supplemented with and without thiamine. Conidia were formed by all transformants on the PD agar without thiamine, and yellowish-green colonies were observed (Figure 3A, upper), whereas few conidia were formed by any strain on the PD agar containing thiamine (Figure 3A, lower). Enumeration of the conidia formed per plate showed that the *Aoatg1* and *Aoatg4* conditional expression strains had a substantial recovery of conidiation, approaching the level of the control strain. In the case of the *Aoatg8* and *Aoatg15* conditional expression strains, however, conidiation was relatively poorly recovered on PD agar compared to the control strain, although an increased number of conidia were observed compared to their gene disruptants (Figure 3B). These results indicate that a sufficient amount of conidia for inoculation are successfully harvested from the *Aoatg* gene-conditional expression strains.

### Repression of *Aoatg* Genes Enhances CHY Production

**Figure 4:**
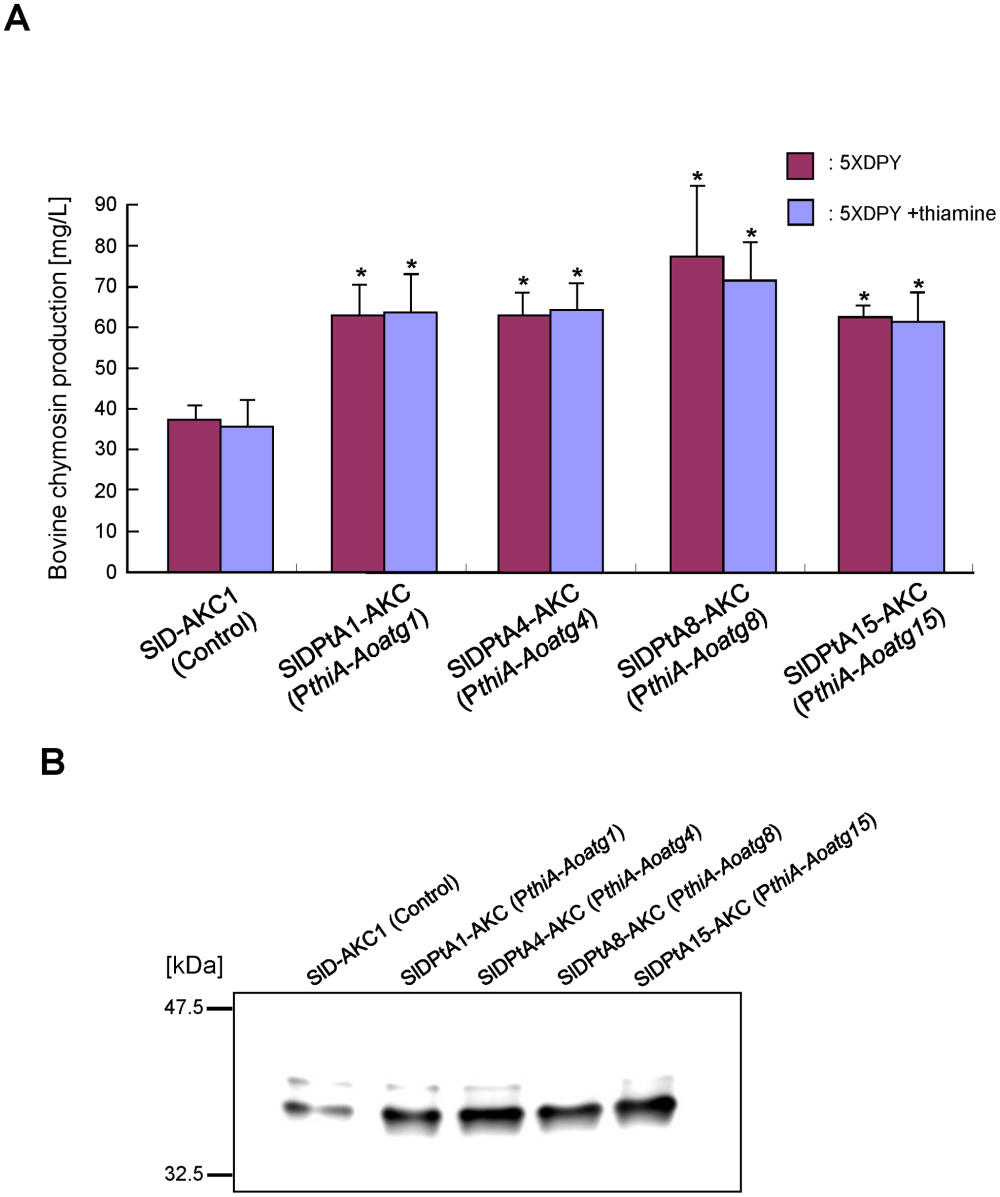
Extracellular bovine chymosin (CHY) production by *A.oryzae* autophagy gene-conditional expression strains. (A) Approximately 2×10^5^ conidia of the control (SlD-AKC1), SlD-PtA1-AKC, SlD-PtA4-AKC, SlD-PtA8-AKC, and SlDPtA15-AKC strains expressing CHY were inoculated into 20 ml 5×DPY medium (pH 5.5) supplemented with and without thiamine. CHY activities in the culture supernatant were measured after 4 days of growth at 30°C. Five experiments were performed, and the values of the average and standard deviations are represented (*p<0.01, Student’s *t* test). (B) Western blot analysis of the culture supernatant of the CHY-expressing strains. Mature CHY bands of 35.4 kDa were detected using an anti-CHY antibody.

To examine heterologous protein production in the *Aoatg1*, *Aoatg4*, *Aoatg8*, and *Aoatg15* conditional expression strains, the CHY expression plasmid was introduced into these strains, and the transformants possessing a single copy of the plasmid, which was integrated homologously at the *niaD* locus were identified by Southern blot analysis (data not shown). The production levels of CHY were then measured (Figure 4 and Figure S2). For each strain, conidia formed on PD agar were harvested and inoculated into 5×DPY medium supplemented with and without thiamine. The CHY production level after 4 days of cultivation in the 5×DPY medium increased by 1.7 fold (*Aoatg1*), 1.7 fold (*Aoatg4*), 2.4 fold (*Aoatg*8), and 1.6 fold (*Aoatg15*) compared to that of the control strain (Figure 4A). Notably, a similar amount of CHY was produced by all strains in the presence and absence of externally supplied thiamine, suggesting that sufficient thiamine to repress the *Aoatg* gene expression was present in the yeast extract component of the 5×DPY medium. Western blotting analysis using an anti-chymosin antibody revealed an increased level of CHY production in the transformants compared to the control strain (Figure 4B). These results suggested that autophagy in the transformants was repressed during submerged culture in the 5×DPY medium.

## Discussion

Autophagy is an intracellular degradation mechanism that delivers cytoplasmic constituents to vacuoles/lysosomes and has recently been shown to play various complex cytological and physiological roles. However, despite the apparent importance of autophagy in eukaryotes, prior to the present study, no reports have examined modification of the autophagic process for enhancing the industrial production of recombinant heterologous proteins. As we predicted that autophagy is a bottleneck for heterologous protein production by *A. oryzae*, we transformed several autophagy gene disruptants with a CHY expression construct and confirmed that the production levels of CHY increased up to 3 fold. However, the poor conidiation by autophagy gene disruptants to be used as hosts for heterologous protein production at the industrial scale is problematic, as sufficient numbers of conidia for inoculation are difficult to obtain. Therefore, we also constructed conditional expression strains of each of the four autophagy genes and demonstrated that the transformants formed increased numbers of conidia, in addition to higher CHY production.

As part of protein quality control in the ER, misfolded proteins are degraded in the ER-associated protein degradation (ERAD) pathway [32]. Autophagy has been reported to serve as another route for ERAD and appears to degrade misfolded proteins that accumulate in the ER lumen [32]. Furthermore, it has been demonstrated that ER stress leads to the induction of autophagy in yeast and mammals [33], [34]. Thus, we speculate that the improvement of CHY protein productivity in most of the *A. oryzae* autophagy gene disruptants was due to impairment of the autophagic-induced degradation of CHY that normally occurs as part of the ER protein quality control process.

In contrast to the *Aoatg1*, *Aoatg4*, *Aoatg8*, *Aoatg13* gene disruptants, the productivity of CHY did not markedly increase in the *Aoatg15* gene disruptant. However, increased CHY production was detected in the *Aoatg15* conditional expression strain. Notably, the *Aoatg15* gene disruptant also displayed the greatest deficiency in the development of aerial hyphae and formation of conidia [29]. Thus, we consider that conidia formed by the *Aoatg15* gene disruptant did not fully mature, because the harvested conidia were white in color, indicating they lacked pigment. In contrast, the conidia recovered from the *Aoatg15* gene-conditional expression strain appeared green in color, and were therefore considered to be fully mature. These results suggest that mature conidia are insufficient to grow of mycelia, which yield an adequate amount of CHY, in the *Aoatg15* disruptant.

In addition, the *Aoatg13* disruptant formed a modest conidia (Figure 1), indicating that autophagic flux was not completely inhibited. This result was consistent with the reported phenotype of these mutants [29], [30]. The disruptant also showed only a slight increase (1.4 fold) in CHY production. These results suggest that the increase of CHY production was correlated with the repression level of autophagy.

In the fission yeast *Schizosaccharomyces pombe*, disruption of multiple protease genes, including *ppp80*/*atg4*, enhances the productivity of human growth hormone (hGH) [35]. However, the *atg4* gene was one of seven protease genes that were disrupted based on a search for such genes in the *S. pombe* genomic database, but the functional effects of *atg4* disruption on hGH production were not examined. In the present study, we revealed that autophagy may have direct effects on heterologous protein production in filamentous fungi by examining CHY in several *A. oryzae* autophagy gene disruptants, including *Aoatg4*, which is involved in autophagosome formation, but also *Aoatg1*, *Aoatg8*, *Aoatg13*, and *Aoatg15*. In addition, the observed correlation between the level of CHY production and repression of autophagy (to the extent that reduction in conidia formation indicates a repression of autophagy) also indicates that autophagy can limit heterologous protein production.

Recently, we identified *Aovps10* encoding an *A. oryzae* vacuolar protein sorting receptor, AoVps10, which is responsible for the recognition and vacuolar sorting of carboxypeptidase Y. Moreover, we demonstrated that the disruption of *Aovps10* in *A. oryzae* leads to enhanced production levels of heterologous proteins [15]. Therefore, it is expected that control of autophagy gene expression, in addition to the disruption of vacuolar protein sorting receptor gene *Aovps10*, will synergistically improve heterologous protein productivity in *A. oryzae*.

Despite the successful enhancement of the extracellular production of CHY by the autophagy gene disruption, there still exist proteolytic problems that should be solved. We previously constructed an *A. oryzae* ten extracellular protease genes disruptant [14]. Thus, another synergy effect of autophagy gene disruptant and the ten protease genes disruptant could be expected. In other words, yields for the heterologous protein production could be efficiently increased when the intracellular (autophagic degradation) and extracellular (proteolysis by proteases) bottlenecks were eliminated.

In conclusion, we successfully constructed *A. oryzae* strains capable of producing high levels of CHY as a model of heterologous protein production due to the repression of autophagy. In addition, to overcome the poor formation of conidia in these strains, the autophagic genes were expressed under control of an inducible promoter. This is the first report to demonstrate that the manipulation of autophagy mechanisms, which have important functions at a basic cellular level, can be applied towards the enhanced production of heterologous proteins. It may be possible to combine this approach for controlling bulk degradation pathways with the disruption of protease genes, which has been demonstrated to be effective [14], [27], for further improving the productivity of heterologous proteins in *A. oryzae*.

## Supporting Information

Figure S1 **Replacement of the** ***Aoatg*** **promoter.** Schemes for the integration of the *thiA* promoter and Southern blotting in *Aoatg1* (A), *Aoatg4* (B), *Aoatg8* (C), and *Aoatg15* (D).
(TIFF)

Figure S2 **Extracellular bovine chymosin (CHY) production by** ***Aoatg8*** **conditional expression strains.** (A) Approximately 2×10^5^ conidia of the control and three individual *Aoatg8* conditional expression strains expressing CHY were inoculated into 20 ml 5×DPY medium (pH 5.5). CHY activities in the culture supernatant were measured after 4 days of growth at 30°C. Three experiments were performed, and the values of the average and standard deviations are represented (*p<0.01, Student’s *t* test).
(TIFF)
