# Supplementary figures and images for: Enhanced Production of Bovine Chymosin by Autophagy Deficiency in the Filamentous Fungus Aspergillus oryzae
Source: PLoS One. 2013 Apr 29;8(4):e62512. doi: 10.1371/journal.pone.0062512 (PMC3639164; doi:10.1371/journal.pone.0062512)

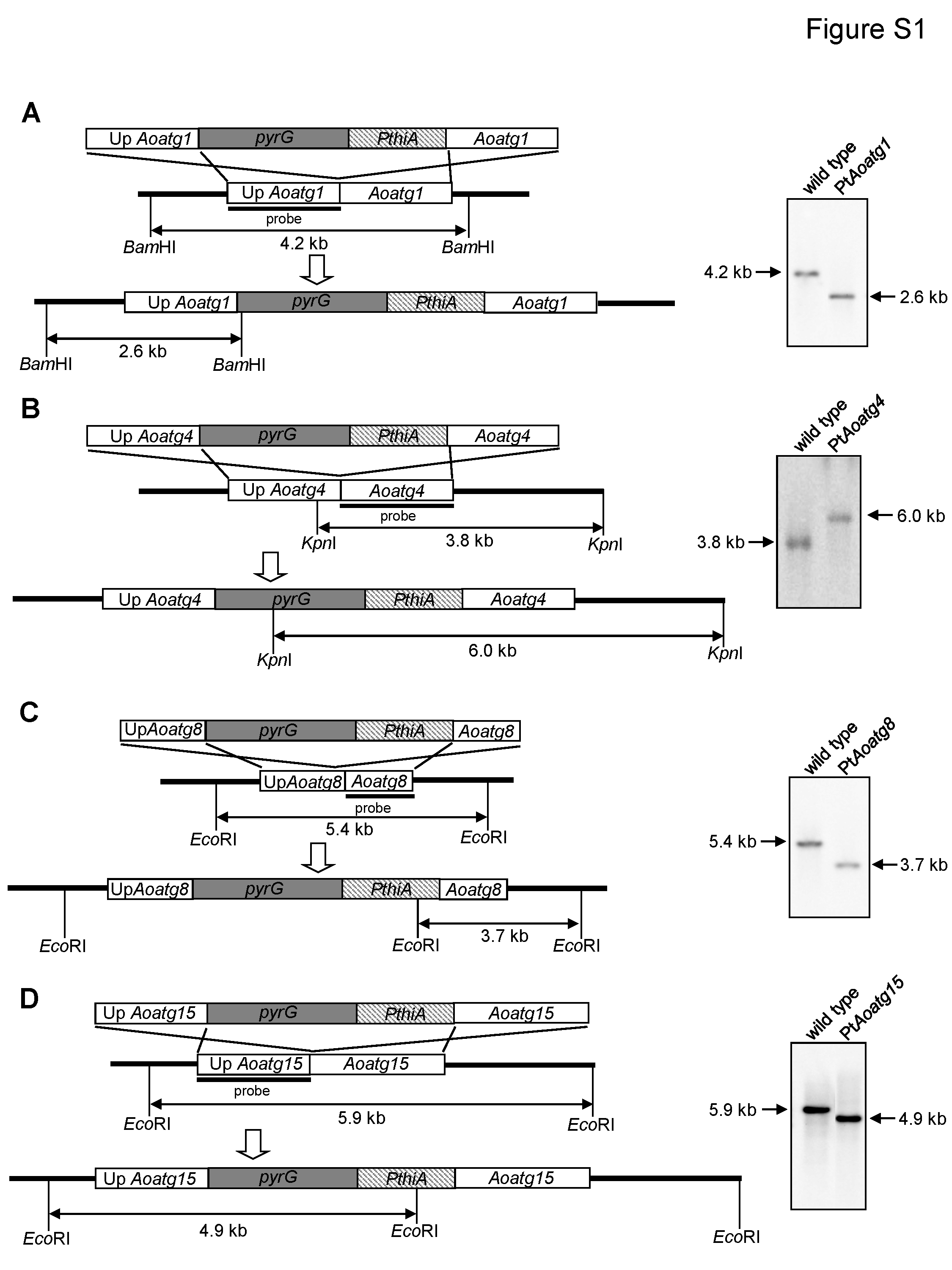

Supplement: Figure S1 — Replacement of the Aoatg promoter. Schemes for the integration of the thiA promoter and Southern blotting in Aoatg1 (A), Aoatg4 (B), Aoatg8 (C), and Aoatg15 (D). (TIFF) [file pone.0062512.s001.tiff]

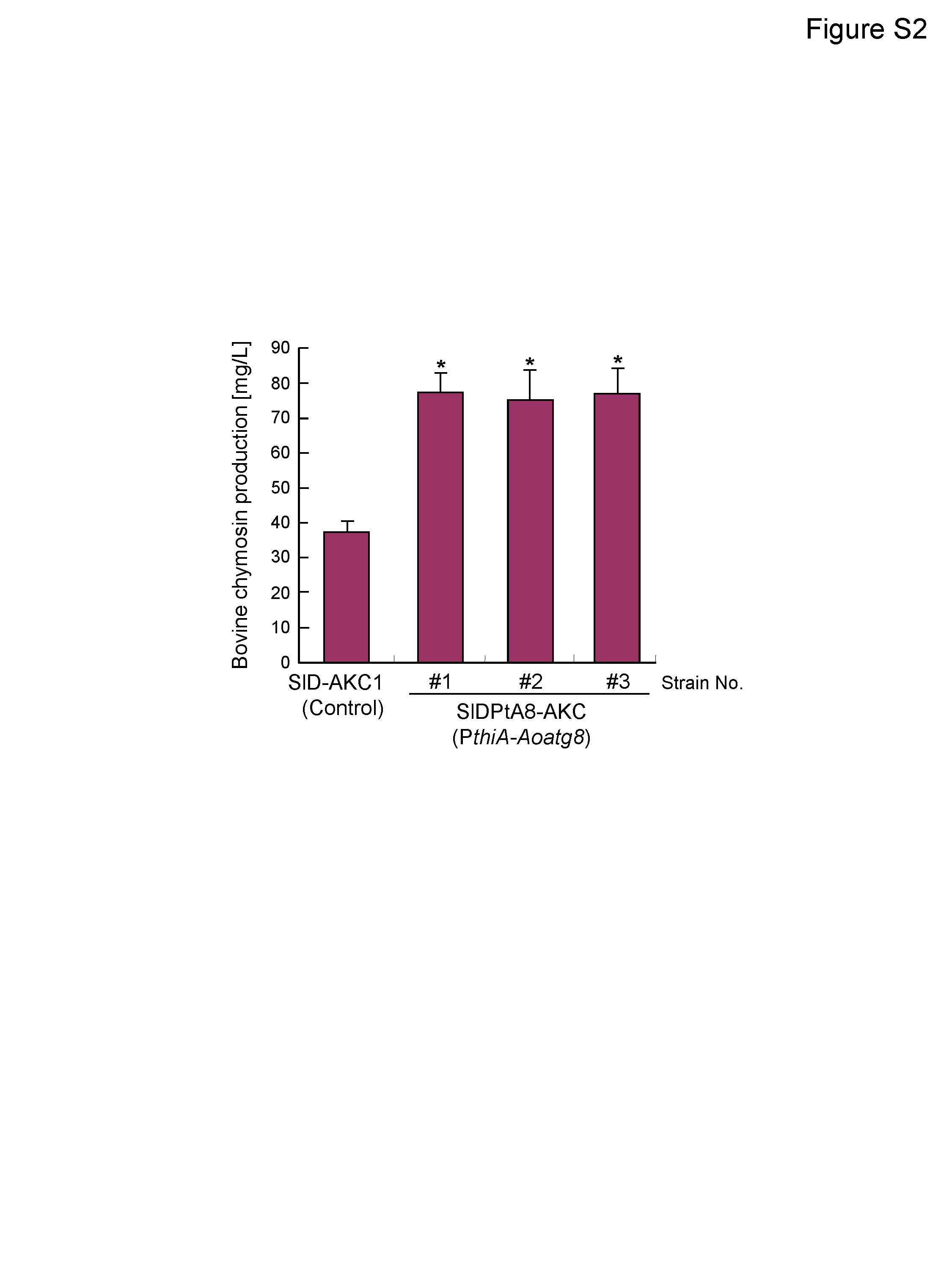

Supplement: Figure S2 — Extracellular bovine chymosin (CHY) production by Aoatg8 conditional expression strains. (A) Approximately 2×10^5^ conidia of the control and three individual Aoatg8 conditional expression strains expressing CHY were inoculated into 20 ml 5×DPY medium (pH 5.5). CHY activities in the culture supernatant were measured after 4 days of growth at 30°C. Three experiments were performed, and the values of the average and standard deviations are represented (*p<0.01, Student’s t test). (TIFF) [file pone.0062512.s002.tiff]
